# *Ginkgo biloba* Extract Inhibits Cisplatin-Induced Acute Kidney Injury-to-Chronic Kidney Disease Through Downregulating Apoptosis Mediated by the HIF-1α/Phosphatidylinositol Pathway

**DOI:** 10.3390/cimb48080834

**Published:** 2026-08-17

**Authors:** Weimin Xu, Ju Huang, Shasha Chen, Yufang Yang, Peiyuan Wan, Xingqing Chen, Xiang Ye, Songqing Huang

**Affiliations:** Pharmacy Department, The First Affiliated Hospital of Guangxi Medical University, Nanning 530021, China

**Keywords:** *Ginkgo biloba* extract, AKI-to-CKD, phosphatidylinositol signaling pathway, HIF-1α, cisplatin, transcriptomics

## Abstract

*Ginkgo biloba* extract (GBe) attenuates the transition of cisplatin (CDDP)-induced acute kidney injury to chronic kidney disease (AKI-to-CKD). Purpose: This study aimed to reveal the mechanism by which GBe inhibits CDDP-induced AKI-to-CKD. The potential targets of GBe in alleviating CDDP-induced renal interstitial fibrosis (Cis-RIF) were predicted through network pharmacology. Transcriptomics and metabolomics were used to detect differentially expressed genes (DEGs) and metabolites (DEMs) in renal tissues from Cis-RIF rats. Integrated multi-omics analysis was performed to determine the potential mechanism underlying GBe inhibiting AKI-to-CKD, and experimental verification was conducted in vivo, in vitro, and using siRNA. We identified 100 targets of GBe that could inhibit Cis-RIF using network pharmacology, and these targets were enriched in 194 signaling pathways. Transcriptomics and metabolomics revealed 8907 DEGs (enriched in 51 pathways) and 424 DEMs (enriched in 16 pathways), respectively. Collectively, the phosphatidylinositol signaling pathway was a co-enriched pathway, which may be the key pathway through which GBe inhibits AKI-to-CKD. This was verified experimentally. The related apoptosis and fibrosis indicators, and the key targets of the phosphatidylinositol signaling pathway (PLC, PKC, PIP2, IP3, DAG, Ca^2+^), in rat renal tissues and renal tubular epithelial cells (RTECs) with CDDP-induced AKI-to-CKD were significantly increased. Inhibition of HIF-1α and knockdown of HIF-1α in RTECs reversed the changes the phosphatidylinositol pathway targets. Moreover, both GBe and the HIF-1α inhibitor could inhibit HIF-1α and the phosphatidylinositol pathway targets, as well as the apoptosis and EMT of RTECs. Conclusion: This study reveals for the first time that GBe may inhibit AKI-to-CKD by downregulating apoptosis and EMT in RTECs through the HIF-1α/phosphatidylinositol signaling axis.

## 1. Introduction

Cisplatin (CDDP) is widely used in the clinical treatment of various tumour types. However, clinically common doses of CDDP can induce acute kidney injury (AKI). AKI can develop into acute kidney injury-to-chronic kidney disease (AKI-to-CKD), which may further progress into chronic kidney disease (CKD), severely restricting the clinical application of CDDP [1,2], and placing a burden on patients and society. Therefore, inhibiting CDDP-induced AKI-to-CKD is of great significance. The mechanisms of CDDP-induced AKI-to-CKD remain unclear, and no effective measures are currently available to prevent and control AKI-to-CKD.

CDDP-induced acute kidney injury (AKI) mainly affects renal tubular epithelial cells (RTECs). Injured RTECs undergo epithelial-to-mesenchymal transition (EMT), a key mechanism driving renal interstitial fibrosis (Cis-RIF) and the eventual progression to chronic kidney disease (CKD) [3,4]. RTEC apoptosis and persistent inflammatory damage are important pathological features of AKI-to-CKD [5,6]. However, the molecular mechanism underlying this process still requires further research. *Ginkgo biloba* leaves (GBe), obtained from dried *Ginkgo biloba* leaves, main contains two types of chemical substances: flavonoid compounds and terpenoid lactones [7]. GBe is known to regulate cell apoptosis, counteract inflammatory damage, and exhibit anti-tumour properties [8,9,10]. We previously reported that GBe can reduce HIF-1α expression in RTECs and inhibit CDDP-induced AKI-to-CKD [11,12]. However, the specific mechanism of action remains unclear.

Network pharmacology has become an effective approach for exploring traditional Chinese medicine (TCM) compounds and their mechanisms of action by constructing drug-target-disease networks [13]. Transcriptomic analyses contribute to the discovery of new pathological mechanisms and therapeutic disease targets [14]. Metabolites are the end products of gene expression and protein function. The conventional apparatus and techniques used in metabolomics analysis can be applied to investigate the functions of genes and proteins, thereby facilitating disease diagnosis and treatment [15,16]. Integrated analysis of network pharmacology, transcriptomics, and metabolomics can reveal the mechanisms by which TCM ameliorates kidney damage [17,18]. In this study, we performed a comprehensive analysis of network pharmacology, transcriptomics, and metabolomics to explore the mechanism by which GBe ameliorates CDDP-induced AKI-to-CKD and verified the findings using in vitro and in vivo experiments.

## 2. Materials and Methods

### 2.1. Drugs and Reagents

*Ginkgo biloba* extract injection (GBe) (lot No: 200-06na095) was obtained from Taiwan Zhonghao International Co., Ltd. (Tai Wan, China). CDDP injection powder (batch No: H20023460) was obtained from Qilu Pharmaceuticals Group Co., Ltd. (Jinan, China). Amphotericin injection powder (AMF) (batch No: H200104030) was purchased from Meiluo Pharmaceutical Co., Ltd. (Dalian, China). Further, 2-methoxyyestradiol (2ME2, a HIF-1α inhibitor) (lot No. s1233) was provided by Selleck Chemicals Co., Ltd. (Houston, TX, USA).

### 2.2. Ethical Statement

The animal experiments in this study were conducted in accordance with the protocols approved by the Institutional Ethics Committee of Guangxi Medical University (No.: 202510016).

### 2.3. Network Pharmacology Analysis of GBe Treatment for Cis-RIF

Network pharmacology analysis was used to probe the mechanisms by which GBe inhibits Cis-RIF. First, the active components of GBe and their corresponding target proteins were retrieved from the Traditional Chinese Medicine System Pharmacology Database, and the relevant targets of Cis-RIF were extracted from the OMIM and GeneCards databases. The targets corresponding to the active components of GBe were then cross referenced with the relevant targets of Cis-RIF to predict the potential targets of GBe in inhibiting Cis-RIF. Finally, using the Metscape database and employing KEGG pathway integration analysis, the signaling pathways related to GBe inhibition of Cis-RIF were identified.

### 2.4. Transcriptomic and Metabolomics Analyses for Renal Tissues from Rats with Cis-RIF

To explore the mechanism underlying the occurrence and development of RIF, transcriptomic and metabolomic analyses were conducted on the renal tissues of Cis-RIF rats. Briefly, male Sprague-Dawley (SD) rats were randomly divided into the Normal (given the same volume of normal saline on Day1) and CDDP groups (intraperitoneally injected with CDDP 5 mg/kg once on Day1) [19]. Specimens were collected after 20 days, and renal function tests, H&E staining, and Masson’s trichrome staining confirmed that the Cis-RIF model was successfully established. Then, transcriptomic and metabolomic analyses were conducted on rat renal tissues from the Normal and CDDP groups [19]. For metabolomic analysis, renal tissue samples were homogenised with solvent (acetonitrile: methanol: water = 2:2:1) at 4 °C, incubated on an ice bath for 1 h, and centrifuged at 4 °C. The supernatant was analysed on an ultra-performance liquid chromatography equipped with quadrupole time-of-flight mass spectrometry (UPLC-Xevo G2-XS QTof-MS system) using the Acquity UPLC^®^ BEH C18 column. The mobile phase consisted of water and acetonitrile with gradient elution at a flow rate of 0.4 mL/min. MS was performed in the positive ion mode. Next, the data were analyzed using the Goa tools database, KOBAS database, and MetaboAnalyst 5.0 platform to identify differentially expressed genes (DEGs) and metabolites (DEMs), as well as their enriched pathways between the Normal and CDDP groups. Finally, common pathways were identified through a comprehensive analysis of transcriptomics and metabolomics.

### 2.5. Integrated Analysis of Transcriptomics, Metabolomics, and Network Pharmacology

To investigate the signaling pathway through which GBe inhibits Cis-RIF, the signaling pathways enriched by the core targets of GBe in Cis-RIF inhibition, as well as the signaling pathways enriched by DEGs and DEMs, were comprehensively analysed, and common signaling pathways were identified.

### 2.6. In Vivo Validation Experiments

Male SD rats were randomly divided into five groups [11]: Control, CDDP, GBe, 2ME2 and AMF. On the first day of the experiment, rats in the CDDP, GBe, 2ME2 and AMF groups were intraperitoneally administered 5 mg/kg CDDP once. From day 8 to day 14, the Control and CDDP groups were intraperitoneally injected with the same volume of normal saline every day, whereas the other groups were intraperitoneally injected with the corresponding drugs, namely 3.17 mg/kg GBe, 4 mg/kg 2ME2, and 1 mg/kg AMF each day. At the end of the experiment, the animals were euthanised by intraperitoneal injection of pentobarbital sodium (30 mg/kg, ip). When the rats were under deep anaesthesia (with relaxed limbs and abdominal muscles, slowed breathing, and disappearance of corneal reflex), blood was collected. After the absence of reflexes and respiration was confirmed, kidney samples were collected and rinsed with chilled normal saline. The induction of AKI-to-CKD in rats by CDDP was confirmed through renal function tests, H&E staining, and Masson staining, and GBe was found to improve renal function and inhibit AKI-to-CKD [11].

This study would examine the apoptosis, fibrosis, and signaling axis-related indicators in the rat renal tissues to explore the mechanism by which GBe inhibits AKI-to-CKD.

### 2.7. Immunohistochemical Detection for α-SMA, Col-1, CTGF, HIF-1α and PKC in Rat Renal Tissues

Rat renal tissues were obtained as described in Section 2.6 “In vivo validation experiments.” Briefly, the kidney sections were dewaxed, dehydrated, and blocked for non-specific binding with 0.3% hydrogen peroxide and 5% BSA buffer. The sections were then incubated overnight at 4 °C with the respective rabbit anti-rat antibodies, including α-SMA (1:500, GB111364), Col-1 (1:1000, GB11022), CTGF (1:300, GB11078), HIF-1α (1:200, 20960-1-AP), PKC (1:1000, GB112251) (all came from Servicebio, Wuhan, China). After incubation with a biotin-labeled secondary antibody (1:100), the sections were subjected to DAB colour development and counterstaining. The positive cells showed blue nuclei and brown cytoplasm. Finally, images were captured using an Olympus microscope. Ten fields of view (×400) were randomly selected in each section, and the positive area, optical density, and IOD values of the acquired images were analysed and measured using Image-Pro analysis software (Image-Pro Plus 6.0).

### 2.8. Ex Vivo Validation Experiments

#### 2.8.1. HK-2 Cell Experiments

Human proximal tubular epithelial cells (HK-2 cells) were purchased from Wuhan Procell Life Science & Technology Co., Ltd. (Wuhan, China). HK-2 cells were divided into six groups and treated with CDDP or GBe (Table 1). After 48 h of the corresponding intervention in each group, HK-2 cells were collected for subsequent testing.

#### 2.8.2. HIF-1α siRNA HK-2 Cell Experiment

To investigate the role of HIF-1α, HIF-1α expression in HK-2 cells was knocked down using siRNA. Briefly, stable HIF-1α knockdown was established in HK-2 cells using lentiviral vectors carrying HIF-1α-specific siRNAs (GenePharma, Shanghai, China), followed by puromycin selection and serial passaging. Knockdown efficiency was verified using western blot and qRT-PCR.

#### 2.8.3. Experimental Study on the Relationship Between HIF-1α and the Key Pathway Targets

HK-2 cells and siHIF-1α HK-2 cells were respectively treated with CDDP (1 μg/mL) for 48 h, and then collected to investigate the effect of HIF-1α on the key targets of the phosphatidylinositol signaling pathway in RTECs after CDDP treatment.

### 2.9. Examination of Apoptosis Using Flow Cytometry

An Annexin V-FITC/PI apoptosis kit was used to detect the apoptosis rate in HK-2 cells. Cells from each group were collected and re-suspended in Binding Buffer, followed by the addition of Annexin V-FITC and PI to each tube. After gentle vortex mixing, the stained cells were incubated at ambient temperature in the dark for 5 min and were analyzed using flow cytometry (BD Biosciences, San Jose, CA, USA).

### 2.10. Western Blot Analysis for Bcl-2, Bax, Caspase 3, α-SMA, Col-1, CTGF, HIF-1α, PLC and PKC in Renal Tissues and HK-2 Cells

Briefly, renal tissues and HK-2 cells were fully lysed using RIPA buffer, and the protein concentration was determined using the BCA assay kit (Beyotime Biotechnology, Shanghai, China). After separation by SDS-PAGE, proteins were transferred to PVDF membranes (Millipore, Billerica, MA, USA). The PVDF membranes were then incubated with specific primary antibodies (4 °C, overnight), including Bax (1:5000), Bcl-2 (1:500), Caspase 3, α-SMA, Col-1, CTGF, HIF-1α (1:500), PLC and PKC. All primary antibodies were obtained from Proteintech Group (Wuhan, China). The primary antibodies were used at a dilution of 1:1000, except for Bax, Bcl-2, and HIF-1α. Finally, after incubation with the fluorescent secondary antibody (goat anti-rabbit, 1:10,000) (CST, Danvers, MA, USA), PVDF membranes were scanned using a near-infrared two-color fluorescence imaging system (Odyssey CLx, LICOR, Lincoln, NE, USA). β-actin was used as the internal standard (1:1000, Proteintech, Wuhan, China).

### 2.11. Quantitative qRT-PCR Analysis for Bcl-2, Bax, Caspase 3, α-SMA, Col-1, CTGF, HIF-1α, PLC and PKC in Renal Tissues and HK-2 Cells

TRIzol reagent (Axygen, California, USA) was used to isolate and extract intact RNA from rat kidney and HK-2 cells. qRT-PCR was performed under the following conditions: 95 °C for 30 s, followed by 40 cycles of 95 °C for 5 s and 60 °C for 31 s. The 2−ΔΔCt method was adopted to analyze the mRNA levels of Bcl-2, Bax, Caspase 3, α-SMA, Col-1, CTGF, and HIF-1α. The specific primers for each gene are listed in Table 2 and Table 3 below.

### 2.12. ELISA Analysis for PIP2, IP3, DAG, Ca^2+^ in Rat Kidney and HK-2 Cells

The levels of PIP2, IP3, DAG and Ca^2+^ in rat kidney, HK-2 cells and HIF-1α siRNA HK-2 cells were measured using ELISA (Elabscience Biotechnology Co, Ltd., Wuhan, China). Rat renal tissues and cells were homogenized using 0.9% sodium chloride solution and then processed according to the kit requirements. The optical density was measured using an enzyme label.

### 2.13. Statistical Analysis

SPSS 26.0 (SPSS Inc., Chicago, IL, USA) was used for statistical analysis in this study. A one-way ANOVA was used for evaluating the disparities among groups. LSD was used for variance homogeneity, and Dunnett’s T3 for variance heterogeneity. Data were expressed as means ± SD. A *p*-value < 0.05 is for statistically significant.

## 3. Results

### 3.1. Signaling Pathway of GBe Inhibiting Cis-RIF Through Comprehensive Analysis of Network Pharmacology, Transcriptomics, and Metabolomics

**Figure 1:**
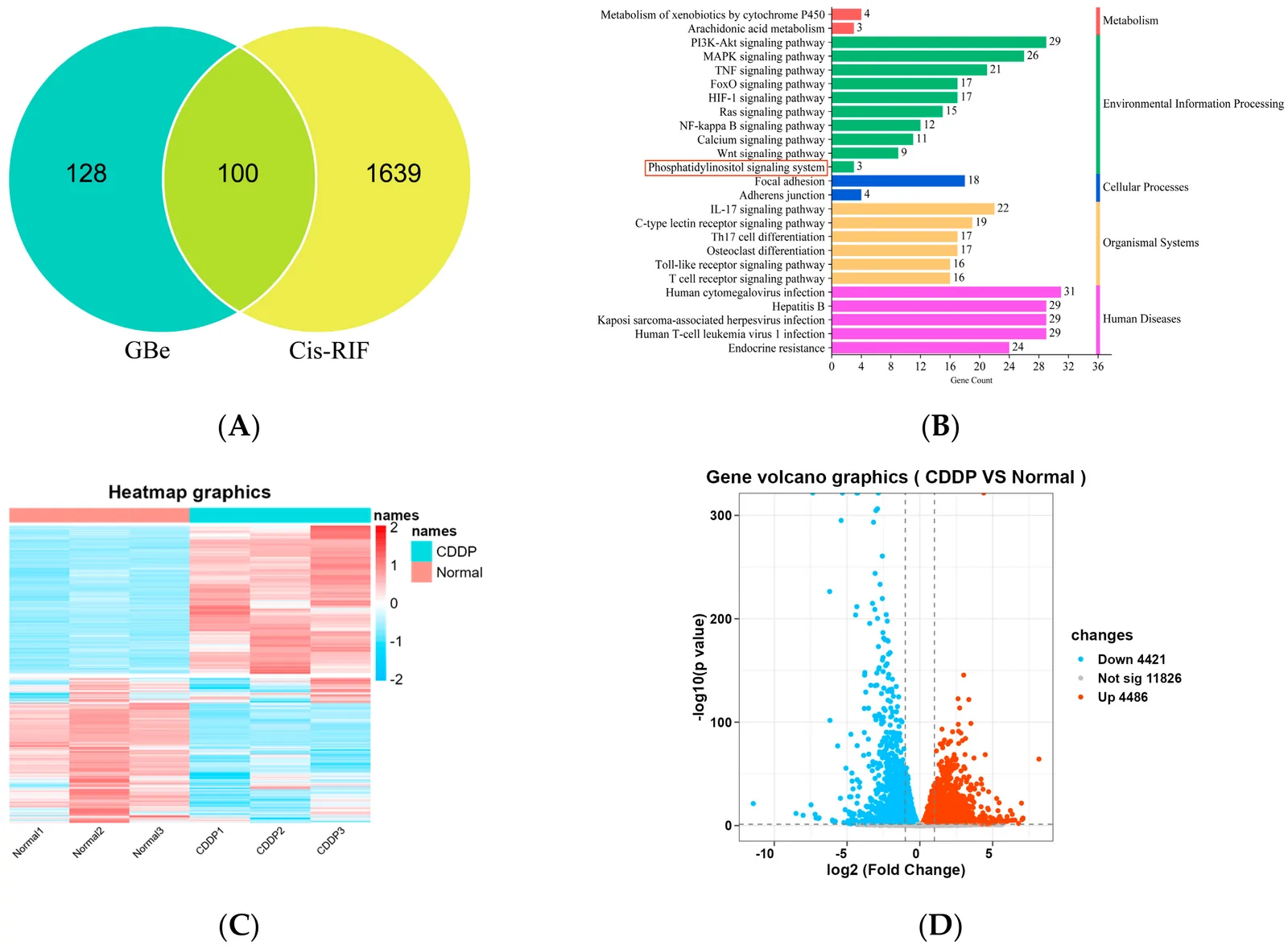

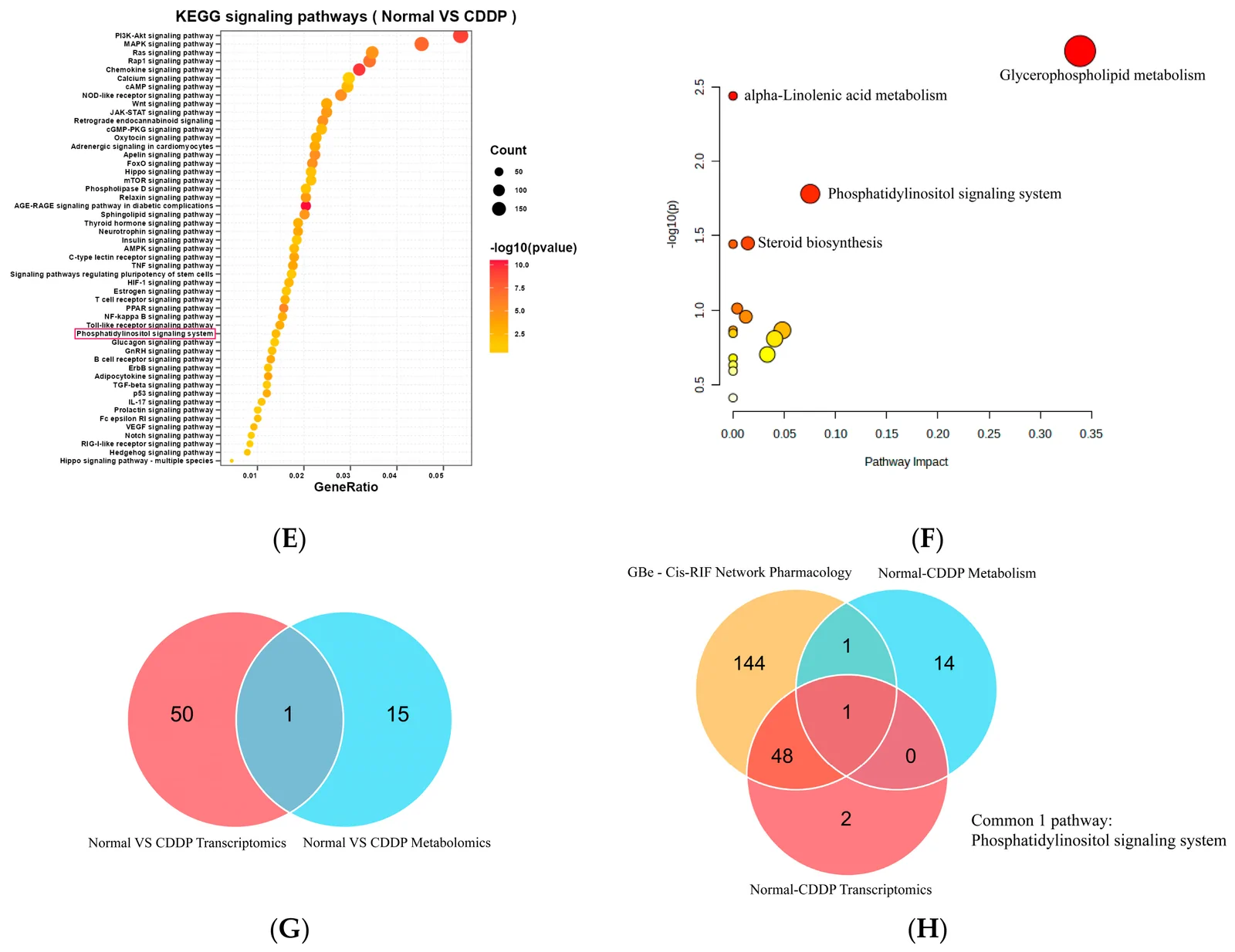
Signaling pathway of GBe inhibiting Cis-RIF was investigated through comprehensive analysis of network pharmacology, transcriptomics and metabolomics. (**A**) Network pharmacological analysis for GBe and Cis-RIF target Venn diagram. (**B**) Network pharmacological KEGG pathway enrichment analysis for potential targets of GBe inhibiting Cis-RIF. (**C**) Heat map of differential expressed genes via transcriptomics. (**D**) Volcano map of gene differential expression via transcriptomics. (**E**) KEGG pathway enrichment analysis of DEGs was performed using Goatools and KOBAS databases. (**F**) Pathway enrichment analysis of differential metabolites using MetaboAnalyst 5.0 platform. (**G**) Transcriptomics and metabolomics Venn diagrams (**H**) Venn diagram of common pathways of network pharmacology, metabolomics and transcriptomics.

Network pharmacology can elucidate the molecular network relationships between diseases and drugs. This approach was used to investigate the mechanism by which GBe inhibits Cis-RIF. First, 228 targets corresponding GBe and 1739 Cis-RIF-related targets were obtained by searching the database. Among these, 100 common targets were identified as potential targets of GBe in Cis-RIF inhibition, and were found to be enriched in 194 signaling pathways through KEGG pathway enrichment analysis (Figure 1A,B).

Next, transcriptomic analysis was performed on renal tissues from rats in the Normal and CDDP groups. The heat map showed the expression patterns of DEGs (CDDP group vs. Normal group) (Figure 1C). Further, the volcano plot revealed 8907 DEGs between the Normal and CDDP groups, including 4486 upregulated genes and 4421 downregulated genes (Figure 1D), which were collectively enriched in 51 signaling pathways (Figure 1E), including the phosphatidylinositol signaling pathway.

Additionally, metabolomic analysis was performed on renal tissues from rats and identified 424 DEMs between the Normal and CDDP groups, which were enriched in 16 metabolic pathways. Among these enriched metabolic pathways, the glycerophospholipid metabolism pathway and the phosphatidylinositol signaling pathway ranked first and second, respectively (Figure 1F).

Finally, by integrating the results of the above network pharmacology, transcriptomics and metabolomics analyses, a common signaling pathway was identified (Figure 1H), namely the phosphatidylinositol signaling pathway. Phosphatidylinositol signaling was thus speculated to be an important mechanistic pathway by which GBe inhibits Cis-RIF.

### 3.2. Effects of GBe on Bcl-2, Caspase 3, Bax, α-SMA, Col-1 and CTGF in the Kidneys

**Figure 2:**
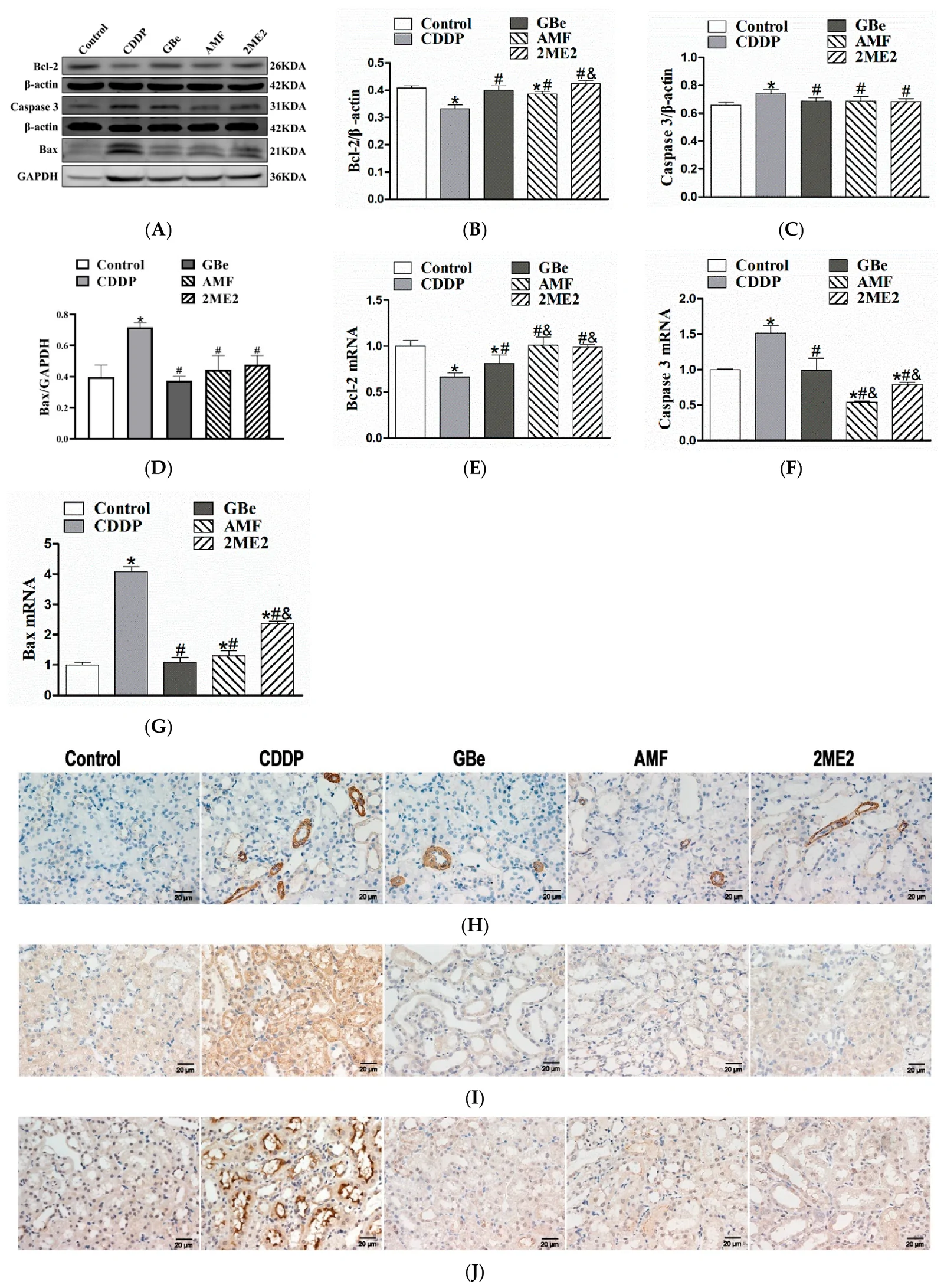

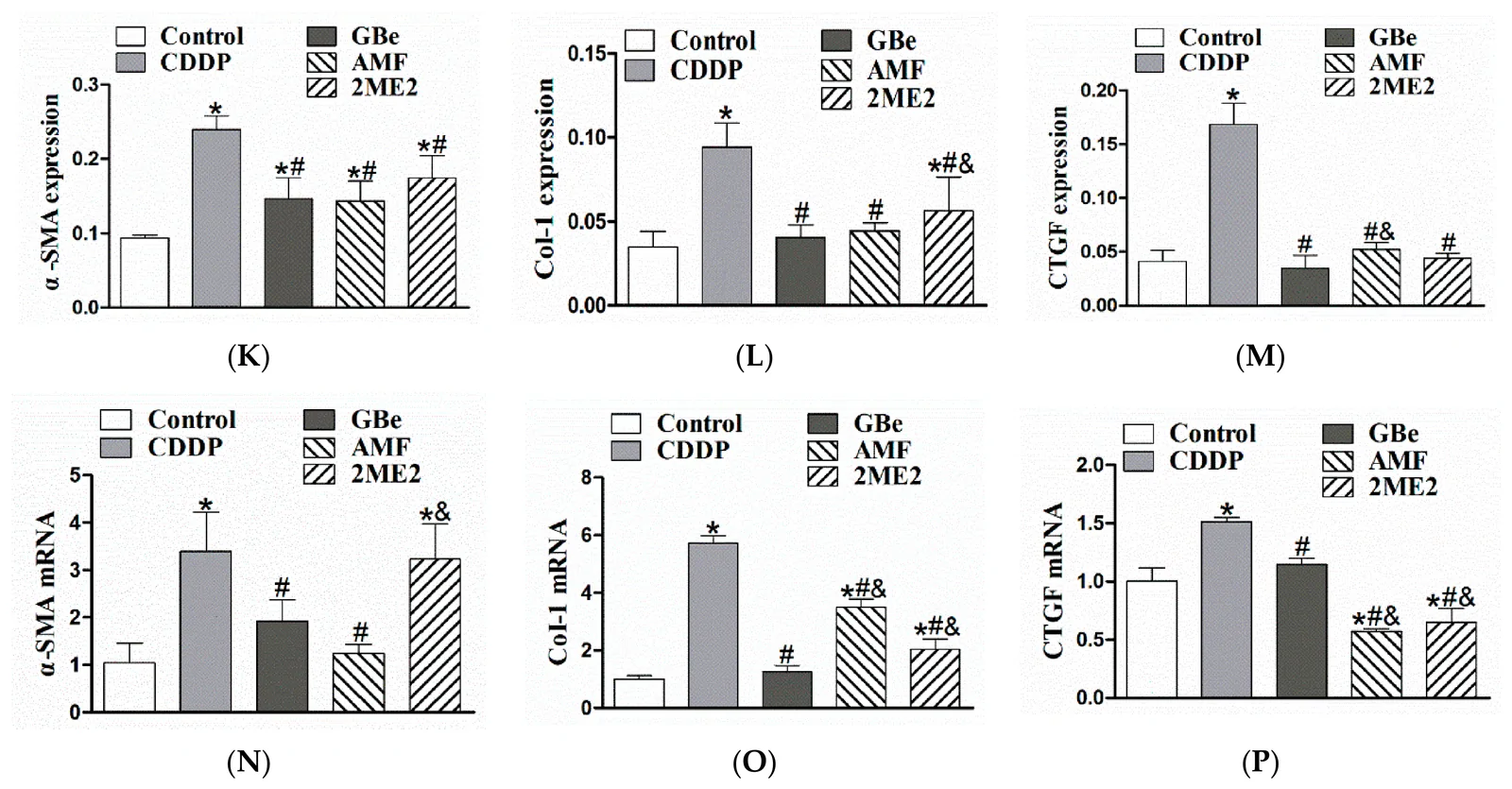
Effect of GBe on the levels of Bcl-2, Caspase 3, Bax, α-SMA, Col-1 and CTGF in kidneys. (**A**–**D**) Western blot analysis for Bcl-2, Caspase 3 and Bax. (**E**–**G**) qRT-PCR analysis for Bcl-2, Caspase 3 and Bax. (**H**–**M**) Immunohistochemical analysis (scale bar = 20 µm) for α-SMA, Col-1 and CTGF. (**N**–**P**) qRT-PCR analysis for α-SMA, Col-1 and CTGF. * *p* < 0.05 vs. Control group, ^#^ *p* < 0.05 vs. CDDP group. ^&^ *p* < 0.05 vs. GBe group.

The protein and mRNA levels of Caspase 3 and Bax were significantly increased, whereas the protein and mRNA levels of Bcl-2 were significantly decreased in renal tissues from rats with CDDP-induced AKI-to-CKD (Figure 2A–G).

Further, the protein and mRNA levels of α-SMA, Col-1, and CTGF were significantly increased in renal tissues from rats with CDDP-induced AKI-to-CKD (Figure 2H–P).

Importantly, GBe could reverse the changes in the above indicators (Figure 2). Notably, the effect of the HIF-1α inhibitor 2ME2 was similar to that of GBe (Figure 2).

### 3.3. Effects of GBe on HIF-1α, PLC, PKC, PIP2, IP3, Ca^2+^ and DAG in Kidneys

**Figure 3:**
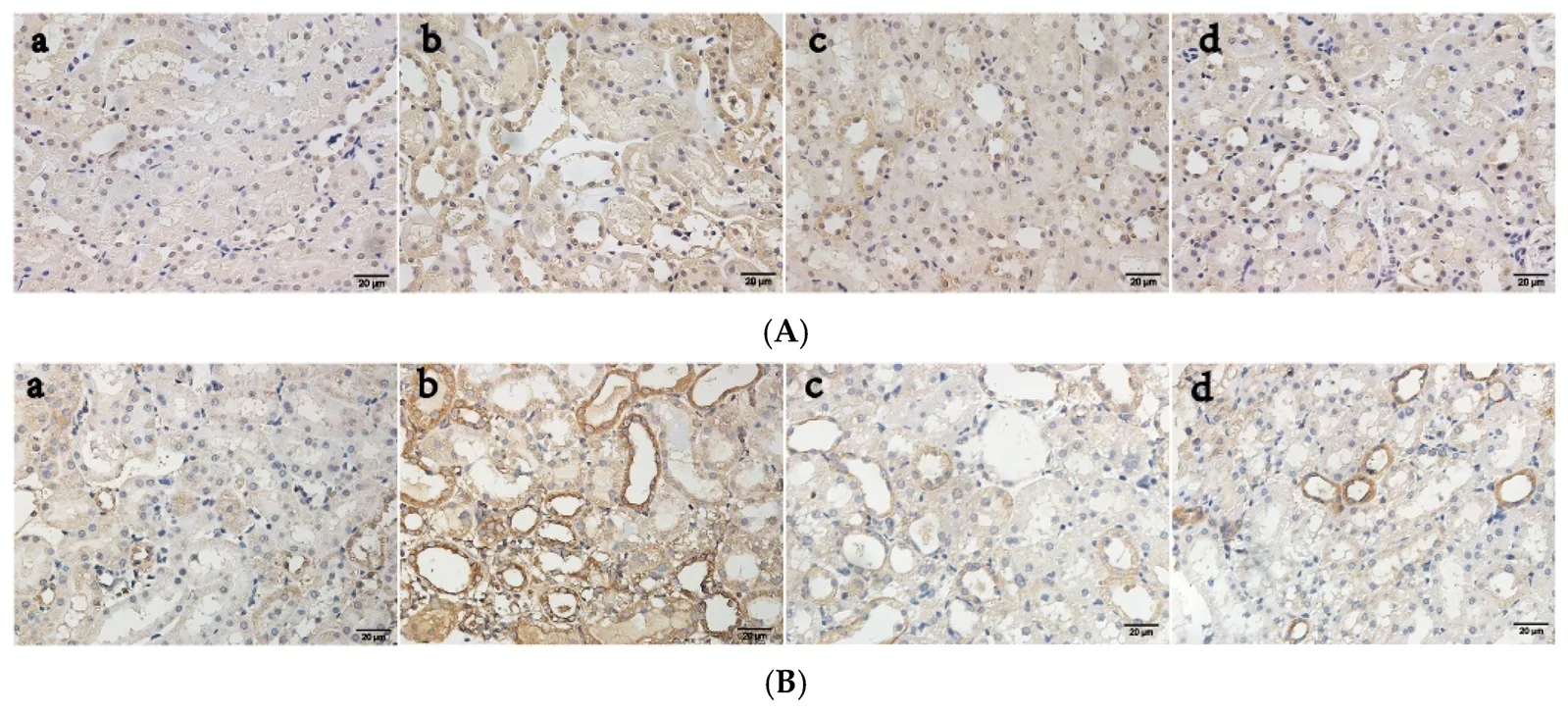

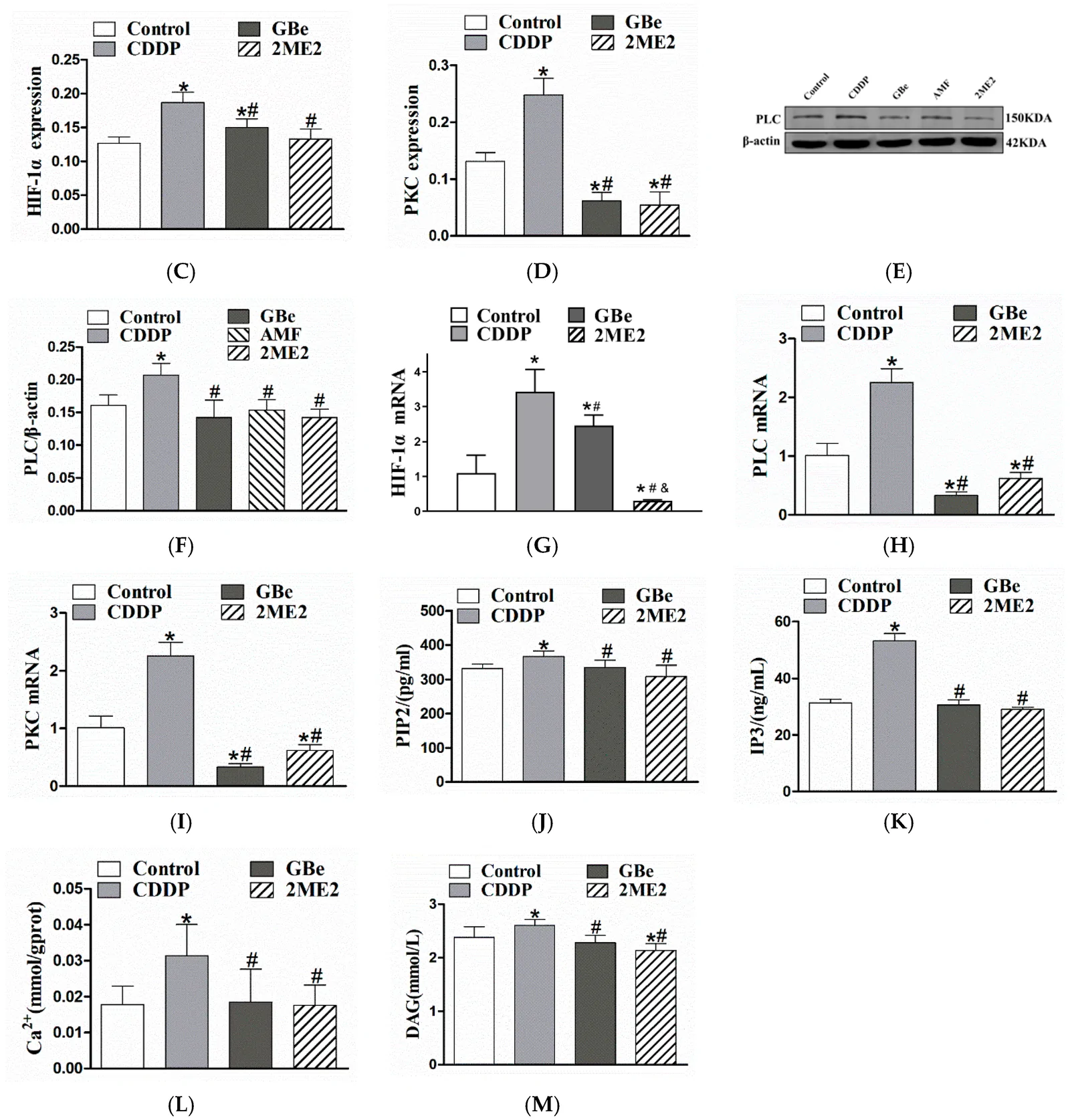
Effects of GBe on the levels of HIF-1α, PLC, PKC, PIP2, IP3, Ca^2+^, DAG in kidneys. (**A**–**D**) Immunohistochemical staining (scale bar = 20 µm) and analysis for HIF-1α and PKC. (**E**,**F**) Western blot analysis for PLC. (**G**–**I**) qRT-PCR analysis for HIF-1α, PLC, PKC. (**J**–**M**) ELISA analysis for PIP2, IP3, Ca^2+^ and DAG. * *p* < 0.05 vs control group, ^#^ *p* < 0.05 vs. CDDP group. ^&^ *p* < 0.05 vs. GBe group. ((**a**): Control group; (**b**): CDDP group; (**c**): GBe group; (**d**): 2ME2 group.).

The protein and mRNA levels of HIF-1α, PLC, and PKC (Figure 3A–I), as well as the levels of PIP2, IP3, Ca^2+^, and DAG, (Figure 3J–M) were markedly increased in kidneys from rats with CDDP-induced AKI-to-CKD. Importantly, both GBe and the HIF-1α inhibitor 2ME2 could significantly decrease the above elevated indicators (Figure 3).

### 3.4. Effects of GBe on HK-2 Cell Apoptosis Rate and Bcl-2, Caspase 3, Bax, α-SMA, Col-1 and CTGF

**Figure 4:**
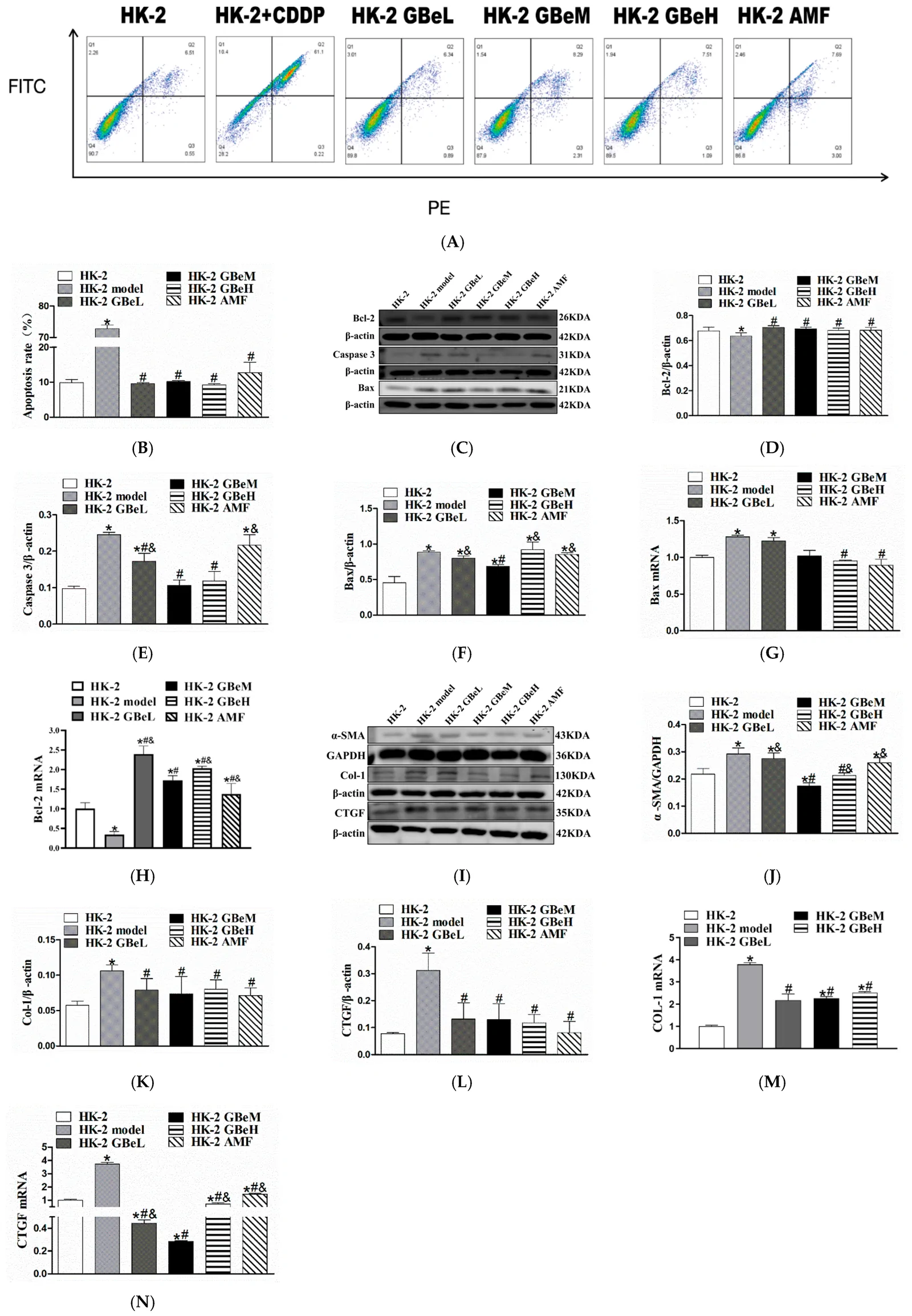
Effects of GBe on apoptosis and EMT in HK-2 cells. (**A**,**B**) Flow cytometry analysis for the apoptosis rate in HK-2 cells. (**C**–**F**) Western blot analysis for Bcl-2, Caspase 3 and Bax. (**G**,**H**) qRT-PCR analysis for Bcl-2 and Bax. (**I**–**L**) Western blot analysis for α-SMA, Col-1 and CTGF. (**M**,**N**) qRT-PCR analysis for Col-1 and CTGF. * *p* < 0.05 vs. HK-2 group, ^#^ *p* < 0.05 vs. HK-2 model group. ^&^ *p* < 0.05 vs. HK-2 GBeM group.

After HK-2 cells were exposed to CDDP, the apoptosis rate, Caspase 3 and Bax protein levels, and Bax mRNA levels in HK-2 cells were significantly increased, whereas both the protein and mRNA levels of Bcl-2 were significantly decreased (Figure 4A–H). Further, the protein levels of α-SMA, Col-1, and CTGF, as well as the mRNA levels of Col-1 and CTGF, were significantly increased after HK-2 cells were exposed to CDDP (Figure 4I–N).

Notably, GBe could significantly reverse the changes in the above indicators in HK-2 cells (Figure 4A–N).

### 3.5. Effects of GBe on HIF-1α, PKC, PLC, PIP2, IP3, Ca^2+^ and DAG in HK-2 Cells

**Figure 5:**
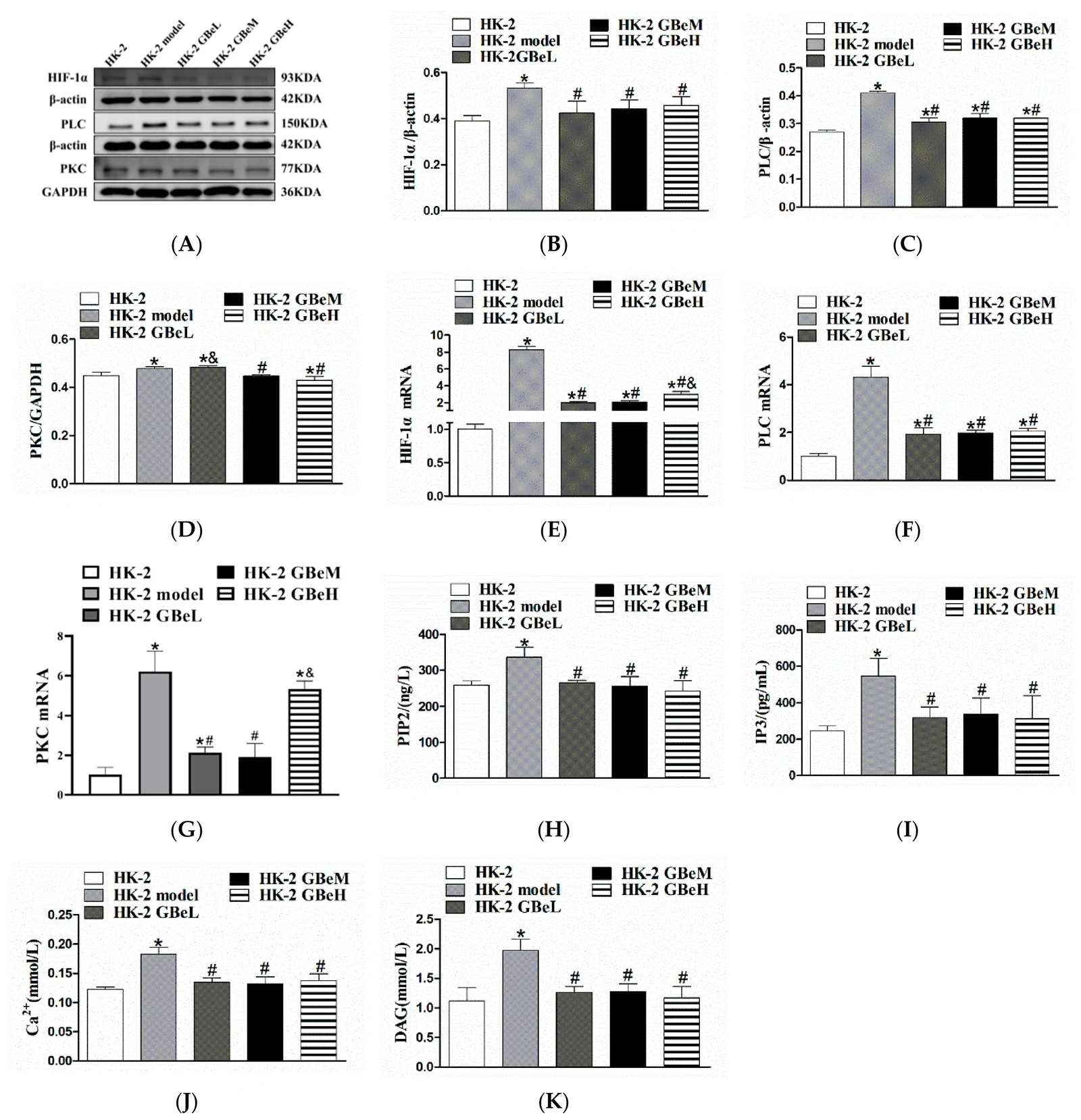
Effects of GBe on levels of HIF-1α, PLC, PKC, PIP2, IP3, Ca^2+^ and DAG in HK-2 cells. (**A**–**D**) Western blot analysis for HIF-1α, PLC and PKC. (**E**–**G**) qRT-PCR analysis for HIF-1α, PLC, PKC. (**H**–**K**) ELISA analysis for PIP2, IP3, Ca^2+^ and DAG. * *p* < 0.05 vs. HK-2 group, ^#^ *p* < 0.05 vs. HK-2 model group. ^&^ *p* < 0.05 vs. HK-2 GBeM group.

As shown in Figure 5, after exposure to CDDP, the protein and mRNA levels of HIF-1α, PLC, and PKC, as well as the levels of PIP2, IP3, Ca^2+^, and DAG, were remarkably increased in HK-2 cells. Importantly, GBe significantly reversed the changes in the above indicators in HK-2 cells (Figure 5).

### 3.6. HIF-1α Regulated the Phosphatidylinositol Pathway in HK-2 Cells

**Figure 6:**
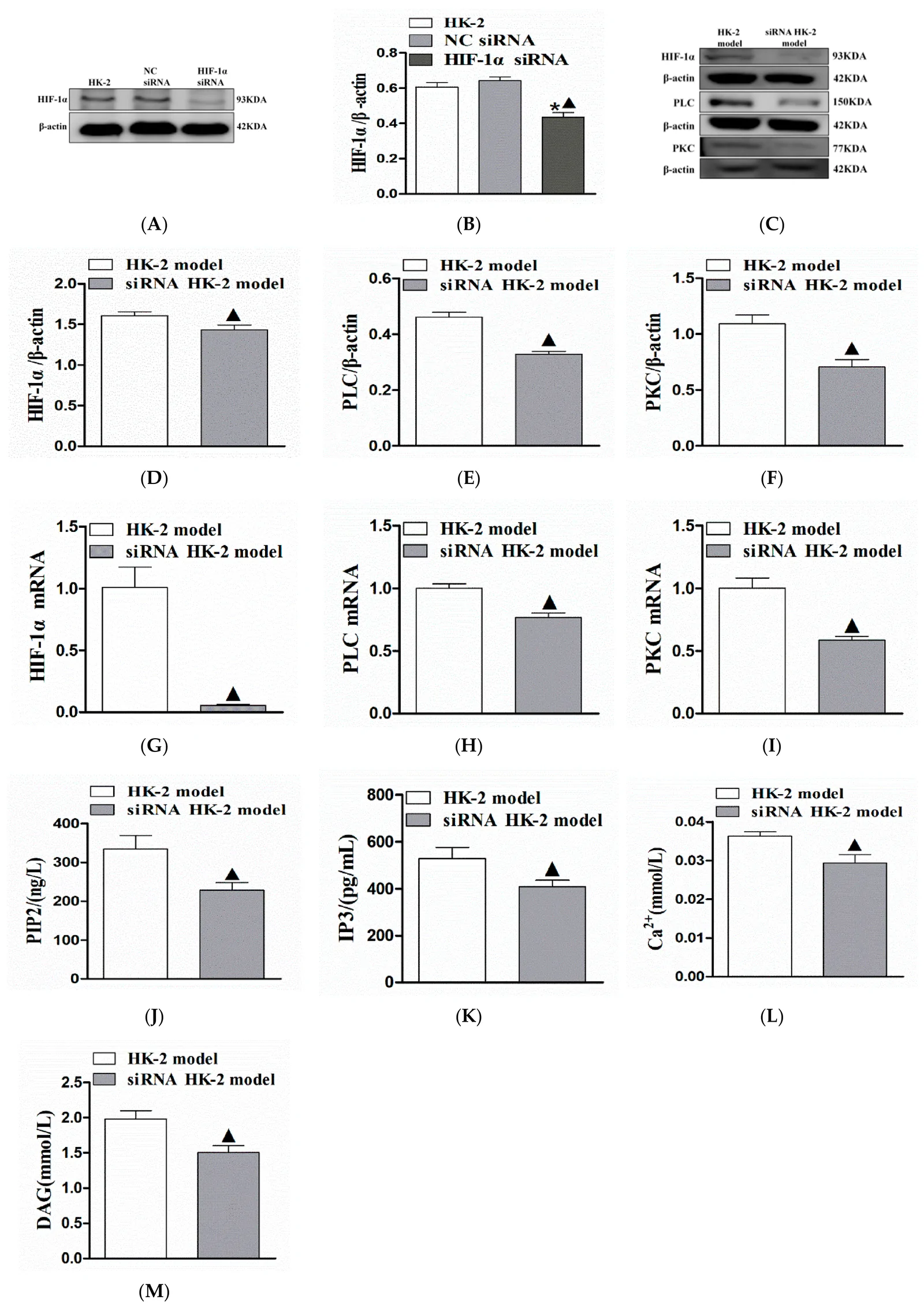
The levels of HIF-1α, PLC, PKC, PIP2, IP3, Ca^2+^ and DAG in HK-2 model group and siRNA HK-2 model group. (**A**,**B**) Western blot analysis for HIF-1α. (**C**–**F**) Western blot analysis for HIF-1α, PLC and PKC. (**G**–**I**) qRT-PCR analysis for HIF-1α, PLC and PKC. (**J**–**M**) ELISA analysis for PIP2, IP3, Ca^2+^ and DAG. * *p* < 0.05 vs. HK-2 group, ▲ *p* < 0.05 vs. HIF-1α siRNA group/HK-2 model group.

As showed in Figure 6A,B, after knocking down HIF-1α expression using siRNA, the HIF-1α protein level in HK-2 cells was significantly decreased, indicating that HIF-1α siRNA HK-2 cells were successfully established.

After exposure to CDDP, the protein and mRNA levels of HIF-1α, PLC and PKC, as well as the levels of PIP2, IP3, Ca^2+^, and DAG, were significantly lower in siRNA HK-2 cells than in HK-2 cells (Figure 6C–M), indicating that HIF-1α could promote the expression of key targets of the phosphatidylinositol signaling pathway in RTECs after CDDP treatment.

## 4. Discussion

CDDP is a first-line clinical anti-tumour drug [20,21], and its efficacy is positively correlated with the dose used. However, the incidence and severity of CDDP-induced nephrotoxicity are also positively correlated with its dose, seriously restricting its clinical application [1,18,22]. Our previous research has shown that a single administration of the conventional clinical dose of CDDP could cause acute kidney injury (AKI) [21]. Over time, RIF can be observed, which continues to deteriorate [10], resulting in CDDP-induced AKI-to-CKD.

CKD is a progressive disease that can progress to chronic kidney failure and, eventually, can only be managed using dialysis or kidney transplantation [23,24]. Therefore, inhibiting the progression of AKI-to-CKD, that is, the occurrence of RIF, is essential [25,26]. However, little is known about the mechanisms underlying the progression of AKI-to-CKD, and effective drugs for prevention and treatment remain insufficient. This study adopted a comprehensive analysis of network pharmacology, transcriptomics and metabolomics, followed by verification through in vivo and in vitro experiments, to explore the role and mechanism of GBe in improving AKI-to-CKD.

GBe is extracted from the leaves of *Ginkgo biloba* and contains many bioactive ingredients, including *ginkgo biloba* flavonoid glycosides [27]. GBe demonstrates anti-apoptotic, anti-inflammatory, blood circulation improving, and neuroprotective functions. Worldwide, GBe has been used to prevent and treat cardiovascular and cerebrovascular diseases, such as stroke, peripheral arterial occlusive disease, disorders of ear or eye blood flow, and nerve disorders [28,29,30]. Recent studies have shown that GBe can inhibit RIF [31]. We have previously found that GBe can alleviate Cis-RIF by inhibiting renal apoptosis [32] and improve AKI-to-CKD by downregulating the HIF-1α pathway in RTECs [11]. However, does GBe inhibit RTECs apoptosis by suppressing HIF-1α and thereby prevent AKI-to-CKD? What is the specific regulatory mechanism? These were the questions that this study aimed to answer.

RIF is well known as a critical pathological change in CKD; RIF severity determines patients’ renal functional reserve, as well as the prognosis of CKD [33,34]. Additionally, an integrated analysis of transcriptomics and metabolomics can clearly identify metabolic changes and further determine the mechanism of disease pathogenesis [35], and a comprehensive analysis of network pharmacology, transcriptomics, and metabolomics can explore the mechanism underlying the multi-target and multi-pathway effects of TCM in disease prevention and treatment [36]. Therefore, this study conducted a network pharmacology analysis and found that the key targets of GBe in inhibiting Cis-RIF were enriched in 194 signaling pathways (Figure 4A,B). Additionally, this study performed transcriptomic and metabolomic analyses and found 8907 DEGs and 424 DEMs in renal tissues from rats with Cis-RIF, which were respectively enriched in 51 and 16 signaling pathways (Figure 4C–F). Finally, the phosphatidylinositol signaling pathway was identified as the common signaling pathway by comprehensively analyzing the above results (Figure 4H). This pathway was considered the key pathway through which GBe inhibits Cis-RIF.

To verify that GBe inhibits CDDP-induced AKI-to-CKD by suppressing the phosphatidylinositol signaling pathway, both in vivo and in vitro experiments were conducted. The results showed apoptosis was significantly increased in the renal tissues of rats with CDDP-induced AKI-to-CKD and in RTECs damaged by CDDP, as confirmed by increased protein and mRNA levels of caspase-3 and Bax, an increased apoptosis rate, and decreased protein and mRNA levels of Bcl-2 (Figure 2 and Figure 4). Meanwhile, renal tissue fibrosis became increasingly severe, and RTECs underwent EMT (Confirmed by the protein and mRNA levels of α-SMA, Col-1 and CTGF significantly increased) (Figure 2 and Figure 4). Importantly, GBe could significantly reverse the abnormal changes in these indicators (Figure 2 and Figure 4). Based on these results, we speculate that GBe may inhibit apoptosis and EMT in RTECs, ultimately suppressing CDDP-induced AKI-to-CKD. However, does GBe regulate apoptosis and EMT in RTECs by modulating the phosphatidylinositol signaling pathway?

The phosphatidylinositol signaling pathway has several important targets, including phospholipase C (PLC), phosphatidylinositol 4, 5-diphosphate (PIP2), inositol 1, 4, 5-triphosphate (IP3), diacylglycerol (DAG), Ca^2+^ and protein kinase C (PKC). PLC can hydrolyse membrane lipid PIP2 into two second messengers, DAG and IP3 [37,38]. IP3 then binds to its receptor (IP3R) on the endoplasmic reticulum, promoting Ca^2+^ release [39] and working together with DAG to activate PKC [40], thereby regulating various cellular functions such as cell apoptosis [41]. However, the role of this pathway in kidney diseases has not yet been reported. This study showed that, the activity of the phosphatidylinositol signaling pathway was significantly increased in renal tissues from rats with CDDP-induced AKI-to-CKD and in RTECs damaged by CDDP (Confirmed by the protein and mRNA levels of PLC and PKC, as well as the levels of PIP2, IP3, DAG and Ca^2+^ were significantly increased) (Figure 3 and Figure 5). Further, GBe could inhibit the activation of the phosphatidylinositol signaling pathway (Confirmed by that GBe reversed the changes of the above key targets of the phosphatidylinositol pathway) (Figure 3 and Figure 5). Based on these results, we conclude that GBe may inhibit apoptosis and EMT in RTECs by regulating the phosphatidylinositol signaling pathway.

To investigate whether HIF-1α affects the phosphatidylinositol pathway, a HIF-1α inhibitor (2ME2) was used in rats with CDDP-induced AKI-to-CKD, and HIF-1α expression in RTECs was knocked down in cell experiments. The results showed that the HIF-1α inhibitor could inhibit the phosphatidylinositol signaling pathway in the kidneys of rats with CDDP-induced AKI-to-CKD, as confirmed by 2ME2 reversing the changes in PLC, PKC, PIP2, IP3, DAG and Ca^2+^ (Figure 3). Additionally, knocking down HIF-1α expression in RTECs could simultaneously reverse the changes in the key targets of the phosphatidylinositol signaling pathway (Figure 6). These results suggest that HIF-1α regulats the phosphatidylinositol pathway. Notably, GBe could significantly reduce the protein and mRNA expression of HIF-1α in the renal tissues of rats with CDDP-induced AKI-to-CKD and in RTECs damaged by CDDP (Figure 3A,C,G and Figure 5A,B,E). These results indicate that GBe can inhibit the HIF-1α/phosphatidylinositol signaling axis.

**Figure 7:**
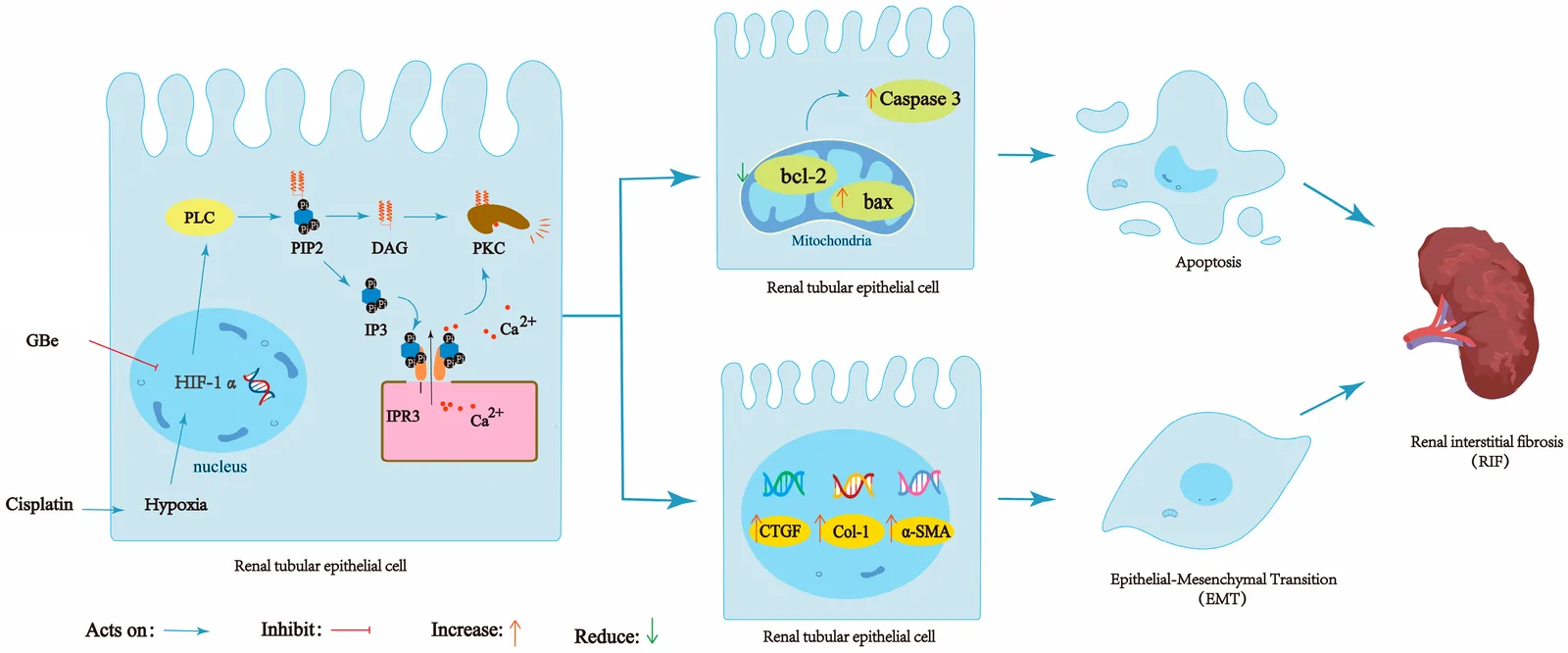
GBe inhibits CDDP-induced AKI-to-CKD by downregulating apoptosis mediated by the HIF-1α/phosphatidylinositol signal axis.

In summary, through a comprehensive analysis of network pharmacology, transcriptomics, and metabolomics, we found that the phosphatidylinositol signaling pathway is the key mechanism by which GBe inhibits CDDP-induced AKI-to-CKD. In vivo and in vitro experiments confirmed that GBe inhibits the HIF-1α/phosphatidylinositol signaling axis, thereby inhibiting apoptosis and EMT in RTECs, and ultimately inhibiting CDDP-induced AKI-to-CKD (Figure 7).

## 5. Innovation

This article explores, for the first time, the mechanism by which GBe improves AKI-to-CKD through a comprehensive analysis of network pharmacology, transcriptomics, and metabolomics. GBe was found to inhibit apoptosis and EMT in RTECs via regulation of the HIF-1α/phosphatidylinositol signaling axis, thereby delaying CDDP-induced AKI-to-CKD.

## Figures and Tables

**Table 1:** Grouping and intervention in HK-2 cell model.

Grouping of Cells	Drug Treatment (48 h)
HK-2	MEM medium (no drugs)
HK-2 model	CDDP (1 μg/mL)
HK-2 GBeL	CDDP (1 μg/mL), GBe (175 μg/mL)
HK-2 GBeM	CDDP (1 μg/mL), GBe (350 μg/mL)
HK-2 GBeH	CDDP (1 μg/mL), GBe (700 μg/mL)
HK-2 AMF	CDDP (1 μg/mL), AMF (0.025 μg/mL)

**Table 2:** List of rat primers used in qRT-PCR for determining mRNA expression.

Gene	Forward Primer (5′-3′)	Reverse Primer (5′-3′)
*Bcl-2*	GTCATGTGTGTGGAGAGCGTC	TCCACAAAGGCATCCCAGCC
*Caspase 3*	ACGCGAAGAAAAGTGACCAT	ACACAAGCCCATTTCAGGGT
*Bax*	CAGGACGCATCCACCAAGAA	GCAAAGTAGAAAAGGGCAACCAC
*α-SMA*	CATCCGACCTTGCTAACGGA	AGTCCAGAGCGACATAGCAC
*Col-1*	CACTGCAAGAACAGCGTAGC	AGTTCCGGTGTGACTCGTG
*CTGF*	GAGGAGTGGGTGTGTGATGAG	GTCTTCCAGTCGGTAGGCAGC
*HIF-1α*	TCTAGTGAACAGGATGGAATGGAG	TCGTAACTGGTCAGCTGTGGTAA
*PLC*	CTTTCTGCGCTTTGTGGTGTATG	TGAGGTCACCATTCTCCTTAGCA
*PKC*	CATCAAGATCACAGACTTCGGCA	CATACAGCAGGACTCCAAAGGAC
*GAPDH*	TGGAGAAACCTGCCAAGTATGATG	TATCCTTGCTGGGCTGGGTG

**Table 3:** List of cell primers used in qRT-PCR for determining mRNA expression.

Gene	Forward Primer (5′-3′)	Reverse Primer (5′-3′)
*Bcl-2*	TTTTTACTCCCTCTCCCCGC	GTCTACTTCCTCTGTGATGTTGT
*Bax*	CCCGAGAGGTCTTTTTCCGA	AGGGCCTTGAGCACCAGTTT
*Col-1*	TCGATGTGGCTCCCTTG	TCTGTTTCCAGGGTTGGG
*CTGF*	TTCCCGAGAAGGGTCAAGC	ATGCCCATCCCACAGGTC
*HIF-1* *α*	CACAGAAGCAAAGAACCCA	GGTGACAACTGATCGAAGG
*PLC*	CATGAGTCCTTCTACTGGCAGC	TGATGTTGTTGCCCGTGATCTG
*PKC*	CTCACTCACCACCCTCAAAA	TGCCCACTGTGTGCCCTTCT
*β-actin* *GAPDH*	CTACCTCATGAAGATCCTCACCGA CACCCACTCCTCCACCTTTGA	TTCTCCTTAATGTCACGCACGATT TCTCTCTTCCTCTTGTGCTCTTGC

## Data Availability

The original contributions presented in this study are included in the article. Further inquiries can be directed to the corresponding author.
